# Integrative Analysis of mRNA and miRNA Expression Profiles of the Tuberous Root Development at Seedling Stages in Turnips

**DOI:** 10.1371/journal.pone.0137983

**Published:** 2015-09-14

**Authors:** Jingjuan Li, Qian Ding, Fengde Wang, Yihui Zhang, Huayin Li, Jianwei Gao

**Affiliations:** Shandong Key Laboratory of Greenhouse Vegetable Biology, Institute of Vegetables and Flowers, Shandong Academy of Agricultural Sciences, Jinan, Shandong, China; Kunming University of Science and Technology, CHINA

## Abstract

The tuberous root of *Brassica rapa* L. (turnip) is an important modified organ for nutrition storage. A better understanding of the molecular mechanisms involved in the process of tuberous root development is of great value in both economic and biological context. In this study, we analyzed the expression profiles of both mRNAs and miRNAs in tuberous roots at an early stage before cortex splitting (ES), cortex splitting stage (CSS), and secondary root thickening stage (RTS) in turnip based on high-throughput sequencing technology. A large number of differentially expressed genes (DEGs) and several differentially expressed miRNAs (DEMs) were identified. Based on the DEG analysis, we propose that metabolism is the dominant pathway in both tuberous root initiation and secondary thickening process. The plant hormone signal transduction pathway may play a predominant role in regulating tuberous root initiation, while the starch and sucrose metabolism may be more important for the secondary thickening process. These hypotheses were partially supported by sequential DEM analyses. Of all DEMs, miR156a, miR157a, and miR172a exhibited relatively high expression levels, and were differentially expressed in both tuberous root initiation and the secondary thickening process with the expression profiles negatively correlated with those of their target genes. Our results suggest that these miRNAs play important roles in tuberous root development in turnips.

## Introduction

The modified tuberous root in many vegetables, such as radishes, carrots, turnips, and sweet potatoes provides a great food source for human consumption. Tuberous roots are rich in nutrition and dietary fiber [1], and convenient for storage. Some are also of medicinal importance [2–3]. Thus, it is important, in the context of economic value, to study the molecular mechanisms underlying the tuberous root formation and development. In additions, since tuberous roots are important modified organs, studies on tuberous root formation and development is also of high biological value.

Tuberous root development is a complex biological process that is controlled by many internal and external factors. Phytohormones, such as auxin [4,5], cytokinin (CTK) [4–6], abscisic acid (ABA) [6], and Jasmonic acid (JA) [7], are key regulators for tuberous formation and development. Auxin and CTK in combination are crucial for tuberous initiation and formation [4–5], while ABA and CTK are likely the main regulation hormones for the secondary thickening growth of tuberous roots [6]. *MADS* box gene *IbMADS1* was proposed to be an important integrator in the JA and CTK-mediated initiation of tuberization [8], while another *MADS* box gene *SRD1* may be involved in the auxin-mediated initial thickening growth of storage roots [4]. *KNOXI* genes (class I Knotted1-like homeobox genes) have a possible function in tuberous root development by regulating CTK levels in tuberous roots of sweet potatoes [9]. *IbEXP1* (an expansin gene) inhibits the initial thickening growth of tuberous roots by suppressing the proliferation of metaxylem and cambium cells [10]. Sucrose is an important factor regulating tuberous root formation and development [11–13]. Sucrose synthase (SuSy) was reported to play an important role in regulating tuberous development [12, 14]. Many environmental factors such as photoperied [15], drought [16], calcium [17], and hypoxia [18] also impact tuberous root development.

Expression studies have been carried out to explore the underlying molecular mechanisms of tuberous root development, based on a variety of techniques, such as simplified differential display analysis [19], suppression subtractive hybridization technology [3], microarray technology [20], and next generation sequencing and large-scale transcriptome analysis [14, 16, 20–23]. According to the expression studies, thousands of genes belonging to hundreds of biological pathways have been identified in tuberous vegetables, such as sweet potatoes [16, 21–23] and radishes [14], indicating that complex molecular networks and pathways are involved in the tuberous root formation and development. Of these pathways, metabolism pathways are likely the dominant ones [14, 21, 23]. Although remarkable progress has been made in the past, the molecular mechanisms are yet to be understood.

MicroRNAs (miRNAs) are a class of 21–24 nucleotide (nt) non-coding RNAs that regulate gene expression at the post-transcriptional level in many important biological processes, including root development [24–28]. It was reported that miR156 and miR172 are involved in the tuberization process of stem in potatoes (*Solanum tuberosum ssp*. *andigena*) [29–30]. Whether miRNAs are involved in the regulation of tuberous root development is still unclear.

Turnip is an important subspecies of *Brassica rapa* with the A genome (2n = 2x = 20, AA), just like Chinese cabbage [31]. Turnip has a modified tuberous root, while Chinese cabbage has a modified leafy head. Both of them are a good system for studying modified organ formation and development. In addition, the genome sequencing of *Brassica rapa* has been completed and the genome size is relatively small and simple, offering an excellent model to study the molecular mechanisms on the whole genome scale. Large-scale transcriptome analyses have been performed on the modified leafy head development of Chinese cabbage at both gene [32] and miRNA levels [33]. However, such work has not been done on the tuberous root of turnips. In this study, we analyzed both mRNAs and miRNAs expression profiles of tuberous roots at different seedling stages in turnips using high-throughput sequencing technology. We identified differentially expressed genes (DEGs), and miRNAs (DEMs) among various developing stages of tuberous roots. The study will lead to a better understanding of the underlying molecular mechanisms that regulate tuberous formation and development in the *Brassica* species.

## Materials and Methods

### Plant materials and growth conditions

The turnip cultivar “Chang Huang Man Jing” was used in this study. The seeds were carefully selected and sown in soil in square pots (8cm in width, length and height, 2 plants/pot) in a greenhouse at 20 ± 2°C under a 16 h light/8 h dark cycle. The plants were watered once a week with the Hoagland solution. All the plants were grown in the same room until collected for sample preparation. Samples consisting of hypocotyl and main root tissues were collected on day 18 (the early stage before cortex splitting, ES), day 28 (the stage of cortex splitting, CSS) and day 64 (the stage of root thickening, RTS) after sowing, respectively. The samples collected from at least 10 seedlings at each stage were pooled together and stored in liquid nitrogen for RNA extraction. All samples were harvested at the same time during the day. To reduce error rates, two independent biological replicates were used for each stage. The samples were labeled as ES1, CSS1, RTS1 for the first biological replicate, and ES2, CSS2, RTS2 for the second biological replicate, respectively.

### RNA isolation

Total RNA was extracted from each sample using Trizol reagent (Invitrogen, Carlsbad CA, USA). The quality of RNA was verified using a NanoDrop ND-1000 Spectrophotometer (Nano-Drop, Wilmington, DE, USA) and a 2100 Bioanalyzer RNA Nanochip (Agilent, Santa Clara, CA, USA), and the quantity of total RNA was determined using the NanoDrop ND-1000 Spectrophotometer. RNA samples were selected for further cDNA library and small RNA (sRNA) library construction according to the following selection criteria: OD260/280≥1.8, OD260/230≥1.8, RIN≥9.0, 28S/18S≥1.6, a concentration of ≥200 ng/μl, and total mass of ≥30 μg. For mRNA sequencing, the total RNA was cleaned up using the RNeasy Plant Mini Kit (74904; Qiagen, Valencia, CA, USA). For sRNA sequencing, RNA fragments of 18–30 nt were isolated and purified from total RNA samples extracted using Trizol reagent after electrophoresing on a denaturing 15% polyacrylamide gel.

### Preparation, sequencing and data processing of mRNA libraries

A total of six mRNA libraries were constructed in this study. The preparation and sequencing of the libraries were performed at Beijing Genomics Institute (BGI, Shenzhen, China) according to the manufacturer's protocol (Illumina). The sample libraries were single end sequenced via an IlluminaHiSeq^TM^ 2000 (Illumina Inc., San Diego, CA, USA). Raw sequence reads were generated and filtered using the Illumina pipeline. Adapters, empty reads, and low quality reads (reads in which the percentage of unknown bases (N) greater than 10%, and reads in which the percentage of the low quality bases with a quality value ≤ 5 greater than 50%) were removed. The high-quality, clean sequences were mapped against the *B*. *rapa* (Chiifu-401) reference genome (http://brassicadb.org/brad/) using SOAPaligner/soap2 [34]. No more than two mismatches were allowed in the sequence alignment. The clean tags mapped to multiple reference genes were filtered, and the remaining tags were designed as unambiguous clean tags. Quality assessment of reads, statistics of alignment, sequencing saturation analysis and randomness assessments were carried out subsequently to assess the quality of sequencing.

We deposited all the sequence data of the six mRNA libraries into NCBI Sequence Read Archive (http://www.ncbi.nlm.nih.gov/sra/), and the accession numbers are SRX850781, SRX852388, SRX852389, SRX852516, SRX852517, and SRX852518 for ES1, ES2, CSS1, CSS2, RTS1, and RTS2 libraries, respectively.

### Identification of DEGs among different seedling stages in the tuberous root of turnips

The gene expression level was calculated using the Reads Per kb per Million reads (RPKM) method [35]. After the gene expression levels of all samples were normalized to RPKM, genes that were differentially expressed among samples were screened according to a strict algorithm [36]. The threshold with a FDR (False Discovery Rate) of ≤0.001 and the absolute value of log2Ratio ≥ 1 was used to assess the significant difference of expression levels of a given gene between two seedling stages. To reduce the false positive rates, only DEGs detected in both biological replicates were considered. To understand the biological functions of DEGs involved in tuberous root development of turnips, Gene Ontology (GO) enrichment analysis using the GO database (http://www.geneontology.org/), and pathway enrichment analysis using the KEGG database (http://www.genome.jp/kegg/) were conducted subsequently.

### Preparation, sequencing and data processing of sRNA libraries

A total of six sRNA libraries were constructed and sequenced at Beijing Genomics Institute (BGI, Shenzhen, China). The sRNA Libraries were prepared following the procedure by Wang *et*. *al* (2013) [33], and were sequenced on an Illumina HiSeq^TM^ 2000. Raw samples of the 49 nt RNA tags obtained from the HiSeq sequencing were cleaned by removing the low quality tags (reads with more than one base with SQ value ≤10 or more than two bases with SQ values ≤13), 5' adaptor contaminants, oversized insertions, no insert tags, poly A tags and small tags (reads shorter than 18 nt). Length distribution of the remaining clean tags was analyzed to determine the compositions of sRNA samples. Subsequently, we mapped the final clean reads against the Chiifu Chinese cabbage genome (http://brassicadb.org/brad/) using SOAP v1.11 to determine gene expression levels and distributions in the genome [34]. The annotation of the clean sRNA tags and prediction of novel miRNAs were performed following the procedure by Wang *et*. *al* (2013) [33]. Putative target genes of the miRNAs were predicted using the rules developed by Allen *et al*. (2005) [37] and Schwab *et al*. (2005) [38].

The reads annotated as rRNA, scRNA, snoRNA, snRNA, and tRNA from GenBank and Rfam were removed, while those annotated as conserved miRNAs from the miRBase database and those predicted as novel miRNAs by Mireap (http://sourceforge.net/projects/mireap/) were used for further analysis in their differential expression profiles among different seedling stages in tuberous roots of turnips.

All the sequence data of the six sRNA libraries were deposited into NCBI Sequence Read Archive (http://www.ncbi.nlm.nih.gov/sra/) under the accession numbers SRX857356, SRX856589, SRX856653, SRX856655, SRX856663 and SRX856672 for ES1, ES2, CSS1, CSS2, RTS1, and RTS2 libraries, respectively.

### Identification of DEMs among different seedling stages in the tuberous root of turnips

The expression level of miRNA in all samples was normalized as transcripts per million (TPM, TPM = actual miRNA count / total count of clean reads × 10^6^). The fold-change and P-value from the normalized expression level were calculated among different samples. The fold change was calculated as log2 Ratio, and the P-value was calculated using the formula described in Wang *et*. *al* (2013) [33]. The threshold with the P-value ≤ 0.05 and an absolute value of log2Ratio ≥ 1 was used to evaluate the significance of the difference of miRNA expression levels. To reduce the false positive rates, only DEMs were detected in both biological replicates were considered.

### Quantitative RT-PCR analysis

To confirm the identified DEGs and DEMs, DEMs and their differentially expressed target genes were subjected to the quantitative RT-PCR (qRT-PCR) analysis. For DEM validation, the first strand cDNA was synthesized using the miRcutemiRNAcDNA Synthesis Kit (DP501, Tiangen, Beijing, China), and qRT-PCR analyses were performed using the miRcutemiRNAqPCR Detection Kit (SYBR Green) (FP401, Tiangen, Beijing, China). Spliceosomal RNA U6 was used as a constitutive expression control. PCR reactions were performed in a 96-well plate at 94°C for 2 min, followed by 45 cycles of reaction (94°C for 20 s, followed by 60°C for 34 s). For DEGs validation, the first strand cDNA was synthesized using M-MLV Reverse transcriptase (M1701, Promega, Madison, USA) and qRT-PCR analyses were performed using a PrimeScript™ RT reagent Kit (Perfect Real Time) (DRR037A, Takara, Dalian, China). PCR reactions were performed in a 96-well plate at 95°C for 10 min, followed by 40 cycles of reaction (95°C for 10 s, followed by 60°C for 60 s). Actin gene was used as a constitutive expression control. All qRT-PCR analyses were performed using an IQ5 Real-Time PCR System (BIO-RAD, Hercules, CA, USA). The specific primers for qRT-PCR are described in S1 Table.

## Results

### mRNA Library construction, sequencing and mapping against the *B*. *rapa* (Chiifu-401) reference genome

To study the global transcriptome during the tuberous root development of turnips, a total of six mRNA libraries (three seedling stages during tuberous root development, two biological replicates for each stage) were constructed and sequenced using an IlluminaHiSeq^TM^ 2000. Approximately 12 million raw tags were generated for each library, and more than 99.0% of the raw tags were identified as clean tags (Table 1). We then mapped all the clean tags against the *B*. *rapa* reference genome (http://brassicadb.org/brad/), and found that over 71% of the clean tags for each library could be mapped onto the reference genes with ≤2 bp mismatch. Less than 29% of the clean tags for each library could not be aligned to any reference genes due to incomplete sequences, and these tags were designated as unknown tags. More than 67.3% (about 8 million) of the clean tags in each library were mapped to a single gene, while less than 4.7% (about 0.5 million) were mapped to multiple reference genes (Table 1). Unknown tags and tags mapped to multi-genes were filtered out, and the unique clean tags mapped to a single gene were reserved for further DEG analysis.

**Table 1:** Statistics of categorization and abundance of tags generated from the six cDNA libraries for DEGs analysis.

Sumary		ES1	CSS1	RTS1	ES2	CSS2	RTS2
Raw tag	Total number	11912091	12059909	12432897	11800705	11847544	12377301
Clean tag	Total number	11808304	11977051	12349827	11686108	11751484	12264388
	Total percent of raw tags	99.13%	99.31%	99.33%	99.03%	99.19%	99.09%
Tag mapping to gene	Total number	8647587	8624710	8864504	8711668	8427102	8762946
	Total percen of clean tags	73.23%	72.01%	71.78%	74.55%	71.71%	71.45%
Unique Tag mapping to gene	Total number	8102741	8116355	8380690	8164325	7924918	8254390
	Total percent of clean tags	68.62%	67.77%	67.86%	69.86%	67.44%	67.30%
Tag mapping to multi-genes	Total number	544846	508355	483814	547343	502184	508556
	Total percent of clean tags	4.61%	4.24%	3.92%	4.68%	4.27%	4.15%
All tag-mapped genes	Total number	30,193	29,963	29,183	30,185	29,737	28,586
	Percent of reference genes	73.33%	72.77%	70.88%	73.31%	72.22%	69.43%
Tag mapping to genome	Total number	9510434	9504974	9759041	9467338	9449714	9770246
	Total percent of clean tags	80.54%	79.36%	79.02%	81.01%	80.41%	79.66%
Unique Tag mapping to genome	Total number	8630156	8711695	8987067	8648126	8530205	8970851
	Total percent of clean tags	73.09%	72.74%	72.77%	74.00%	72.59%	73.15%
Tag mapping to multi-position of genome	Total number	880278	793279	771974	819212	919509	799395
	Total percent of clean tags	7.45%	6.62%	6.25%	7.01%	7.82%	6.52%
Unknown tag	Total number	3160717	3352341	3485323	2974440	3324382	3501442
	Total percent of clean tags	26.77%	27.99%	28.22%	25.45%	28.29%	28.55%

The saturation for the six libraries was analyzed, and the results showed that the number of detected genes increased with the number of reads until the number of reads reached up to 2 million (S1 Fig), which indicates that the sequencing depth is sufficient for the transcriptome coverage in this study. We also assessed the randomness of RNA fragmentation in the six libraries to estimate whether it was sufficient for a subsequent bioinformatics analysis of gene expression, and the results showed that the reads in each position of the reference gene were distributed evenly and demonstrated highly similar tendencies in all libraries (S2 Fig).

### Differentially regulated genes in tuberous root development of turnips

**Fig 1:**
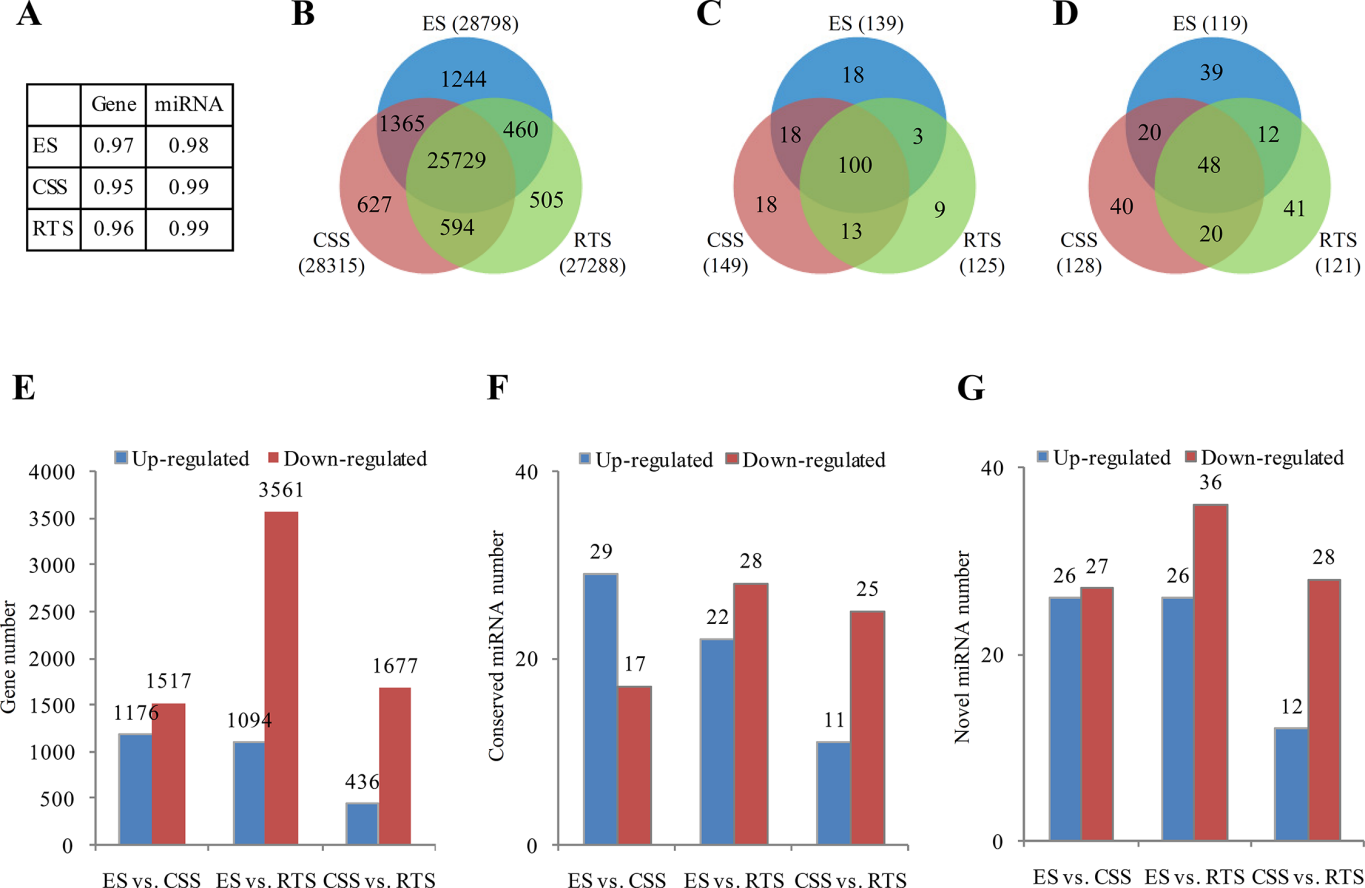
Characterization of gene and miRNA expression patterns among different stages of tuberous root in turnips. (A) Correlation coefficients of two biological duplicates; (B) Venn diagram analyses of stage specific genes in tuberous root of turnips; (C) Venn diagram analyses of stage specific conserved miRNA in tuberous root of turnips; (D) Venn diagram analyses of stage specific novel miRNA in tuberous root of turnips; (E) Differentially expressed genes across all libraries; (F) Differentially expressed conserved miRNAs across all libraries; (G) Differentially expressed conserved novel miRNAs across all libraries.

Based on deep sequencing of the six mRNA libraries, approximately thirty thousand genes (about 70% of reference genes in Chinese cabbage) were detected in each library (Table 1, S2 Table). To compare gene expression differences in different stages of tuberous root development in turnips, the gene expression level was calculated using the RPKM method [35]. Gene expression correlations based on RPKM between the two biological replicates were analyzed, and the correlation coefficients (*R* ^*2*^) were high (0.97, 0.95, and 0.96 for ES, CSS, and RTS, respectively) (Fig 1A). Genes expressed in both replicates in the same stage were screened. 28,798, 28,315, and 27,288 genes were expressed in both replicates in ES, CSS, and RTS, respectively. Most of them (25,729) were expressed in all three stages, while only a small portion (460–1,365) was expressed at two stages or specifically at one stage (Fig 1B).

We compared the gene expression profiles between every two stages in tuberous root development in both biological replicates (ES1 vs. CSS1, ES1 vs. RTS1, CSS1 vs. RTS1, ES2 vs. CSS2, ES2 vs. RTS2, and CSS2 vs. RTS2, where the former is the control and the latter is the experimental group). The results are presented in S2 and S3 Tables. DEGs detected in both biological replicates were screened for subsequent analysis. A total of 2693 (1176 up- and 1517 down-regulated), 4655 (1094 up- and 3561 down-regulated), and 2133 (436 up- and 1677 down-regulated) genes were differentially expressed in both replicates in the stage pairs, ES vs. CSS, ES vs. RTS, and CSS vs. RTS, respectively (Fig 1E). More genes, which were down-regulated, were detected than the up-regulated ones in all pairs (Fig 1E).

**Fig 2:**
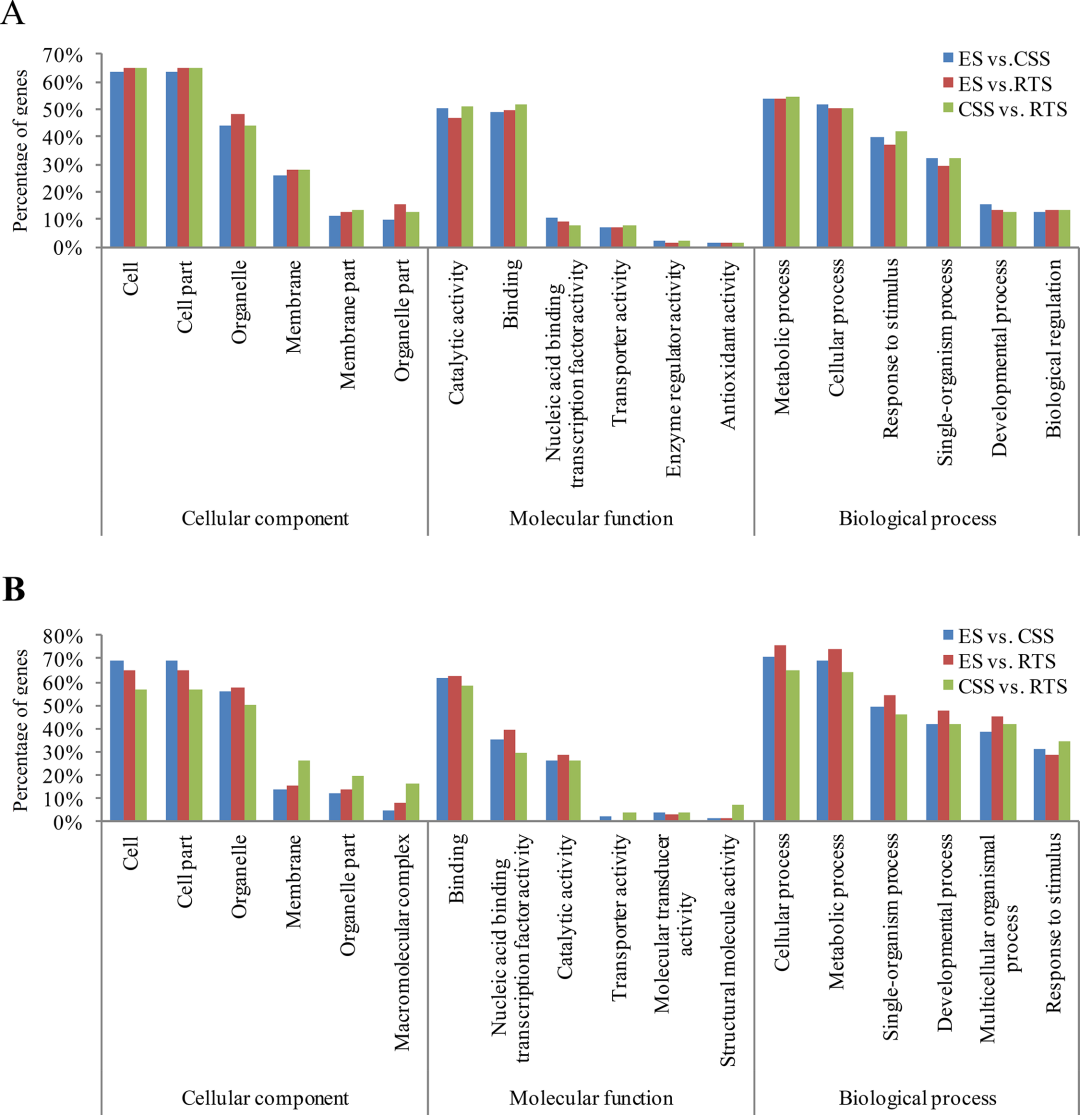
Gene classification based on gene ontology (GO) for DEGs and target genes of DEMs during tuberous root development. (A) Gene classification based on GO for DEGs. Top six classes are shown for cellular component, molecular function and biological process, respectively; (B) Gene classification based on GO for target genes of DEMs. Top six classes are shown for cellular component, molecular function and biological process, respectively.

Gene Ontology (GO) enrichment analysis of the DEGs was performed to gain insight into the possible mechanisms involved in tuberous root development. All the well-annotated DEGs were functionally clustered into three main groups (cellular component, molecular function, and biological process). For cellular component ontology, a large number of the DEGs in the stage pair, ES vs. CSS, involved in cell, cell part, and organelle, accounted for 63.73%, 63.73%, and 44.15% of all the well-annotated DEGs, respectively. Under the molecular function category, catalytic activity (50.26%) and binding (48.62%) were the main groups, followed by nucleic acid binding transcription factor activity (10.54%) and transporter activity (6.88%) in ES vs. CSS. For biological process category, the main groups of the DEGs in ES vs. CSS were involved in metabolic process (53.74%), cellular process (51.42%), response to stimulus (39.89%), and single-organism process (32.31%). Similar results were obtained for GO enrichment analysis of the DEGs in ES vs. RTS, and CSS vs. RTS (Fig 2).

We performed pathway enrichment analysis by mapping all the annotated genes to terms in the KEGG database to identify significantly altered biological pathways associated with the tuberous root development in turnips. The results are shown in Table 2. A total of 17, 20, and 21 pathways were significantly enriched with Q value ≤0.05 in ES vs. CSS, ES vs. RTS, and CSS vs. RTS, respectively. Of these, 11 pathways were significantly enriched in all comparison pairs, indicating that these pathways may be involved in both tuberous root initiation and the secondary thickening process. Four pathways, including plant hormone signal transduction, amino sugar and nucleotide sugar metabolism, other glycan degradation, and cutin, suberine and wax biosynthesis, were only enriched in ES vs. CSS, implying the great importance of these pathways in tuberous root initiation. The plant hormone signal transduction pathways were significantly enriched in ES vs. CSS. Among these seven phytohormones, namely auxin, CTK, brassinosteroid (BR), gibberellin (GA), JA, ethylene, and ABA, signal transduction pathway of auxin had the most DEGs (S4 Table). Metabolic pathways, biosynthesis of secondary metabolites, and plant hormone signal transduction were the top three enriched pathways in ES vs. CSS, while metabolic pathways, biosynthesis of secondary metabolites, and starch and sucrose metabolism were the top three enriched pathways in both ES vs. RTS and CSS vs. RTS (Table 2).

**Table 2:** Pathway enrichment analysis of differentially expressed genes (Top ten enriched pathways are shown).

	Pathway	DEGs withpathway annotation	All genes with pathway annotation	Qvalue
ES vs. CSS	Metabolic pathways	453	4992	9.70E-08
	Biosynthesis of secondary metabolites	328	2757	8.14E-20
	Plant hormone signal transduction	162	1555	8.05E-06
	Starch and sucrose metabolism	84	622	2.33E-07
	Phenylpropanoid biosynthesis	83	456	1.50E-13
	Pentose and glucuronate interconversions	59	356	3.13E-08
	Stilbenoid, diarylheptanoid and gingerol biosynthesis	55	321	3.13E-08
	Phenylalanine metabolism	49	223	4.71E-11
	Limonene and pinene degradation	39	242	1.55E-05
	Amino sugar and nucleotide sugar metabolism	33	278	2.06E-02
ES vs. RTS	Metabolic pathways	892	4992	6.56E-12
	Biosynthesis of secondary metabolites	524	2757	1.92E-10
	Starch and sucrose metabolism	145	622	4.71E-08
	Phenylpropanoid biosynthesis	96	456	9.16E-04
	Pentose and glucuronate interconversions	85	356	2.19E-05
	Stilbenoid, diarylheptanoid and gingerol biosynthesis	68	321	6.76E-03
	Photosynthesis	63	111	7.52E-23
	Phenylalanine metabolism	58	223	5.41E-05
	Limonene and pinene degradation	53	242	1.04E-02
	Carbon fixation in photosynthetic organisms	52	153	2.35E-08
CSS vs. RTS	Metabolic pathways	366	4992	8.95E-05
	Biosynthesis of secondary metabolites	214	2757	3.58E-04
	Starch and sucrose metabolism	64	622	2.53E-04
	Phenylpropanoid biosynthesis	46	456	3.44E-03
	Stilbenoid, diarylheptanoid and gingerol biosynthesis	42	321	4.26E-05
	Phenylalanine metabolism	34	223	1.89E-05
	Limonene and pinene degradation	33	242	1.52E-04
	Ascorbate and aldarate metabolism	26	146	1.89E-05
	Alanine, aspartate and glutamate metabolism	21	144	1.48E-03
	Arginine and proline metabolism	21	182	1.83E-02

### sRNA Library construction, sequencing and mapping against the *B*. *rapa* reference genome

sRNA libraries from the six samples of turnips were sequenced using an Illumina HiSeq^TM^ 2000 to reveal the global expression profiles of miRNAs involved in tuberous root development of turnips. Over ten million raw reads were generated from each library. More than 99.4% of the raw reads were identified as clean reads in each library. A total of 3,819,650, 4,555,962, 4,164,927, 4,090,180, 4,665,954, and 4,130,002 unique sRNAs were obtained after collapsing redundancies from the clean reads of the ES1, CSS1, RTS1, ES2, CSS2, and RTS2 libraries, respectively (Table 3). The length distribution of the clean reads in each library was summarized, and the results showed that 24 nt sRNAs were the most abundant in all six sRNA libraries, accounting for 40.53%-51.54% of all sRNAs. The 21 nt class was the second most abundant in the ES1, RTS1, and ES2 libraries, while the 23 nt class was the second most abundant in the CSS1, CSS2, and RTS2 libraries (S3 Fig). We mapped the final clean sRNAs against the reference Chinese cabbage genome (http://brassicadb.org/brad/), and found that 66.72%- 68.96% of the total clean sRNAs and 50.27%- 51.72% of the unique sRNAs of the six libraries could be matched with the reference genome (Table 3). The percentages of mapped total clean sRNAs and unique sRNAs were similar in all of the six sRNA libraries.

**Table 3:** Statistics of the sRNA sequences generated from the six libraries.

Summary		ES1	CSS1	RTS1	ES2	CSS2	RTS2
Raw reads	Total number	12055844	12900016	10921688	11181610	12429678	10153805
Clean reads of total sRNAs	Total number	11971710	12773948	10842660	11103933	12328840	10076719
	Total percentage of raw tags	99.58%	99.46%	99.72%	99.59%	99.62%	99.68%
Genome-matched total sRNAs	Total number	8256257	8668954	7292990	7531225	8310526	6723439
	Total percentage of clean tags	68.96%	67.86%	67.26%	67.82%	67.41%	66.72%
Unique sRNAs	Total number	3819650	4555962	4164927	4090180	4665954	4130002
Genome-matched unique sRNAs	Total number	1920234	2327847	2145992	2073703	2406889	2135972
	Total percentage of unique sRNAs	50.27%	51.09%	51.53%	50.70%	51.58%	51.72%
Total miRNAs	Total number	2230799	1126436	585858	1652860	1037822	542609
	Total percentage of total sRNAs	18.63%	8.82%	5.40%	14.89%	8.42%	5.38%
Unique miRNAs	Total number	17465	21972	14890	13422	16983	14769
	Total percentage of unique sRNAs	0.46%	0.48%	0.36%	0.33%	0.36%	0.36%

### Identification of conserved and novel miRNAs in the tuberous root of turnips

The clean sRNAs were annotated following the procedure described in Wang *et al*. (2013) [33], and the distribution of sRNAs among different categories is shown in S5 Table. A total of 17,465 (0.46%), 21,972 (0.48%), 14,890 (0.36%), 13,422 (0.33%), 16,983 (0.36%), and 14,769 (0.36%) candidate miRNAs were identified from the ES1, CSS1, RTS1, ES2, CSS2, and RTS2 libraries, respectively (S5 Table). We aligned all these miRNA candidates to the miRNA precursor/mature miRNA of all plants deposited in the miRBase database to identify conserved miRNAs. Only perfectly matched or closely related (≤ 2 mismatches) sequences and precursors that could form hairpins were defined as conserved. A total of 173, 187, 162, 165, 179, and 157 conserved miRNAs were identified from the ES1, CSS1, RTS1, ES2, CSS2, and RTS2 libraries, respectively. In all, 279 conserved miRNAs were identified in this study, among which, 98 were expressed in every library (S6 Table). Conserved miRNAs expressed in both replicates in the same stage were screened for further analysis. It was found that 139, 149, and 125 were expressed in both replicates in ES, CSS, and RTS, respectively (Fig 1C). One hundred of the total detected conserved miRNAs were expressed in all three stages, while 2–18 were only expressed at two stages or specifically at one stage (Fig 1C).

Currently, 43 miRNAs (35 miRNAs, 4 miRNA-5p and 4 miRNA-3p) in *B*. *rapa* have been deposited in the miRBase database (miRNA Registry, Release 20.0, 2013). In this study, 38, 36, 31, 39, 35, and 31 bra-miRNAs were identified from the ES1, CSS1, RTS1, ES2, CSS2, and RTS2 libraries, respectively (S7 Table). Collectively, 42 of these 43 bra-miRNAs deposited in the miRBase database, except bra-miR5723, were identified in at least one of the six libraries; 27 bra-miRNAs were found in each of the six libraries. However, over half (23–26) of these 43 bra-miRNAs (e.g. bra-miR5720, bra-miR5717, and bra-miR5724) showed a very low level (less than 100 reads) or no expression in all libraries, while only 3–6 (e.g. bra-miR157a and bra-miR164a) were expressed dramatically high (more than 5000 reads) in all or most of the libraries (S7 Table). This finding indicates that the expression of conserved miRNAs varies significantly in the tuberous root of turnips. Bra-miRNAs expressed in both replicates in the same stage were screened, and 36, 34, and 29 were expressed in both replicates in ES, CSS, and RTS, respectively (S7 Table).

Novel miRNAs were predicted by exploring the secondary structure, the Dicer cleavage site and the minimum free energy of the unannotated sRNA tags which could be mapped to the genome using the Mireap program developed by BGI (http://sourceforge.net/projects/mireap/). Accordingly, 292, 292, 279, 266, 287, and 266 novel miRNAs were identified in the ES1, CSS1, RTS1, ES2, CSS2, and RTS2 libraries, respectively (S6 Table). Most of the novel miRNAs (more than 85%) showed very low expression level (less than 100 reads), and only three (novel_mir_14, novel_mir_176, and novel_mir_26) showed an expression level higher than 1000 reads in all six libraries. A total of 912 novel miRNAs were identified, but only 48 were expressed in all six libraries (S6 Table), indicating significant variation of novel miRNA expression in different samples. A total of 119, 128, and 121 were expressed in both replicates in ES, CSS, and RTS, respectively (Fig 1D). Venn diagram analyses of stage specific novel miRNAs in tuberous root of turnips are shown in Fig 1D.

### Differentially regulated miRNAs in tuberous root development of turnips

miRNA expression correlations between the two biological replicates were analyzed according to TPM. The correlation coefficients (*R* ^*2*^) were 0.98, 0.98, and 0.98 for ES, CSS, and RTS, respectively (Fig 1A). Based on the criteria described in the Methods section, DEMs between different stages in each replicate were identified, and the detailed results are listed in S6 Table. DEMs detected in both the biological replicates were screened for subsequent analysis. In all, 46 (29 up- and 17 down-regulated), 50 (22 up- and 28 down-regulated) and 36 (11 up- and 25 down-regulated) conserved miRNAs (Fig 1F), and 53 (26 up- and 27 down-regulated), 62 (26 up- and 36 down-regulated) and 40 (12 up- and 28 down-regulated) novel miRNAs (Fig 1G) were differentially expressed in both replicates in ES vs. CSS, ES vs. RTS and CSS vs. RTS, respectively. Eight miRNAs (miR156a, miR157a, miR157a-3p, miR5718, novel_mir_4, novel_mir_85, novel_mir_142, and novel_mir_236) were differentially expressed in both replicates of all comparison pairs, indicating that these miRNAs may be involved in both tuberous root initiation and the secondary thickening process. Twenty miRNAs were differentially expressed in ES vs. CSS, implying a potential role of these miRNAs in tuberous root initiation (S6 Table).

We found 128 (73 for 20 conserved DEMs and 67 for 22 novel DEMs), 163 (97 for 26 conserved DEMs and 66 for 23 novel DEMs), and 97 (53 for 12 conserved DEMs and 44 for 13 novel DEMs) putative target genes for DEMs in ES vs. CSS, ES vs. RTS, and CSS vs. RTS, respectively (Table 4, S8 Table). GO enrichment analyses of the target genes of DEMs (both conserved and novel DEMs) detected in both replicates were conducted subsequently. For cellular component ontology, cell, cell part, and organelle were the main groups of the target genes; under the molecular function category, binding, nucleic acid binding transcription factor activity, and catalytic activity were the main groups; for the biological process category, cellular process, metabolic process, and single-organism process were the top three groups (Fig 2B). The results of GO analysis in the target genes of DEMs were similar to those of DEGs in this study (Fig 2).

**Table 4:** Statistics of target genes of DEMs.

		ES vs. CSS	ES vs. RTS	CSS vs. RTS
Conserved miRNAs	miRNA number	20	26	12
	target gene number	73	97	53
	number of targets per miRNA	3.65	3.73	4.42
Novel miRNAs	miRNA number	22	23	13
	target gene number	67	66	44
	number of targets per miRNA	3.05	2.87	3.38

Pathway enrichment analyses of the target genes of DEMs detected in both replicates were also performed using the KEGG pathway database. No pathway was significantly enriched with Q value of ≤0.05 in all the three comparisons, which may be because of the small numbers of the target genes. More genes were enriched in plant hormone signal transduction pathway in ES vs. CSS and ES vs. RTS, while more were enriched in metabolic pathways in CSS vs. RTS (S9 Table).

The expression profiles of the target genes of DEMs were analyzed according to the high-throughput sequencing of mRNA. The results showed that a number of DEMs had target gene(s) differentially expressed in the DEG analysis. Some differentially expressed target genes had their expression profiles matched up with those of their corresponding DEMs (S8 Table). These DEGs and DEMs are the most important candidates for further study of their functions in tuberous root development of turnips.

### Validation of DEGs and DEMs by qRT-PCR

**Fig 3:**
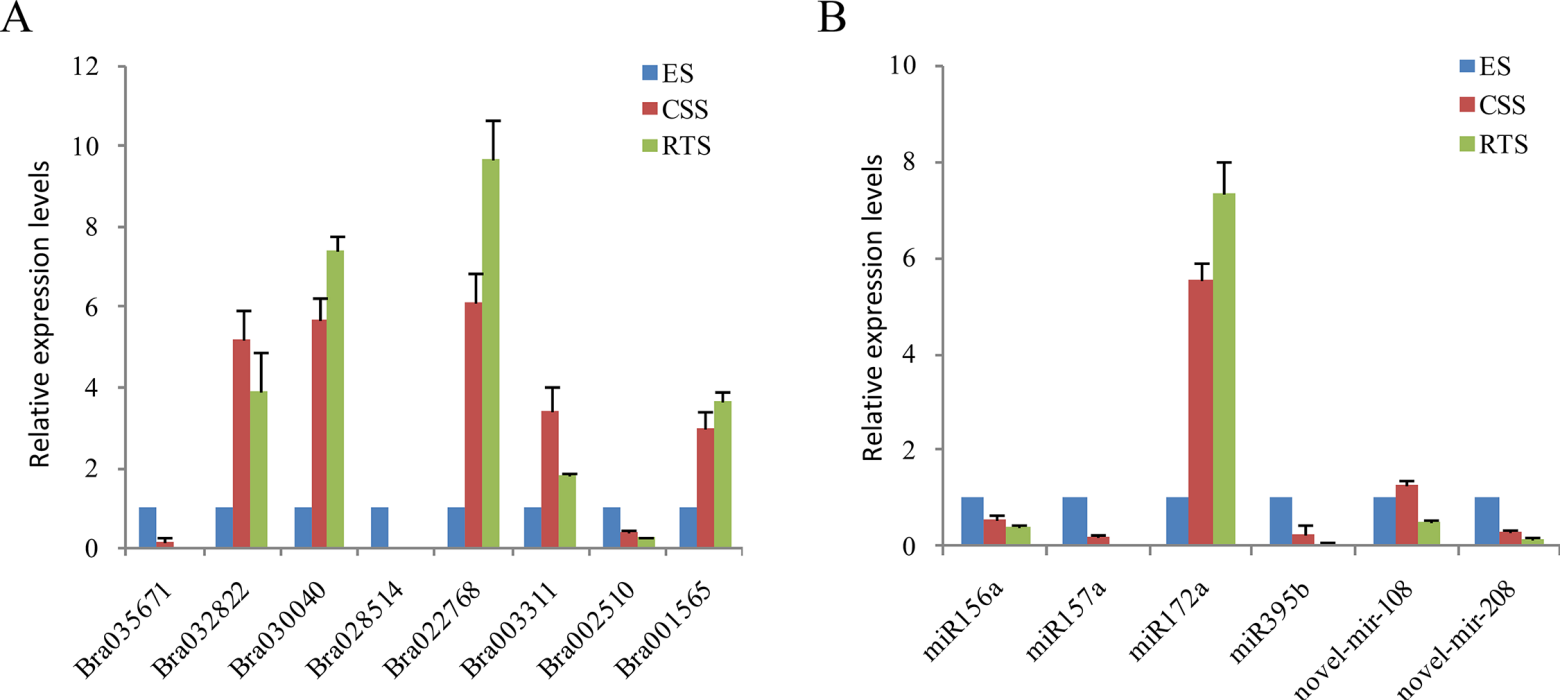
qRT-PCR validation of DEGs and DEMs from high-throughput sequencing analyses. (A) qRT-PCR validation of DEGs from high-throughput RNA sequencing analyses. Relative expression levels were calculated using actin2 as the reference gene using the formula 2^-△△Ct^. The values indicate means of three biological replicates ± standard error. (B) qRT-PCR validation of DEMs from high-throughput sRNA analyses. Relative expression levels were calculated using spliceosomal RNA U6 as the reference gene using the formula2^-△△Ct^. The values indicate means of three biological replicates ± standard error.

The expression profiles of six DEMs between different stages and their seven differentially expressed target genes were verified by qRT-PCR. The results showed that all seven genes and four miRNAs (miR156a, miR157a, miR172a, and novel-mir-208) exhibited the same expression tendency as the original results (Fig 3, S2 and S6 Tables). The expression profile of miR395b did not match with that of the original results (Fig 3B, S6 Table), probably due to its low expression level. Similarly the expression profile of novel-mir-108 did not match either. Of the four miRNAs exhibiting the same expression tendency as the original results, miR156a, miR157a, and miR172a exhibited relatively high expression level in all samples, while novel-mir-208 expressed extremely low in all samples in the sRNA sequencing.

## Discussion

### Quality assessment of sequencing

Tuberous root development is a complex biological process controlled by interactions of genetic, physiological and environmental factors. High-throughput sequencing, owing to its advantages of high coverage and low cost, has been widely used to study complex biological processes of plants in recent years. Here, in order to gain insight into the underlying molecular mechanisms regulating tuberous root development, large-scale analyses of both mRNA and miRNA expression profiles of tuberous roots at different seedling stages in turnips were performed by high-throughput sequencing technology. Good repeatability was achieved, according to the high correlation coefficients (*R* ^*2*^>0.95, Fig 1A) between the two replicates on both mRNA and miRNA expression levels. The number of raw reads, the percentages of clean tags, and the percentages of clean tags mapped onto the reference genome in both mRNA and miRNA expression profile analyses were greater than, or similar to, those in previous studies in the brassica species [33, 39–44]. According to the results of sequencing saturation analysis and randomness assessment, the clean tags obtained in this study were sufficient for subsequent bioinformatics analyses of gene expression. The size distribution of sRNAs in each sRNA library was also similar with those in previous studies in Chinese cabbage [33, 42–47]. However, there were some small differences between different libraries. The 21nt sRNAs were the second most abundant class in CSS libraries, while the 23 nt sRNAs were the second most abundant class in RTS libraries [S3 Fig], implying the stage specific functions of these sRNAs in the process of tuberous root development in turnips. In ES libraries, the second major classes of sRNAs were not the same in the two replicates [S3 Fig], which is probably due to sequencing errors.

### Genes and biological pathways involved in tuberous root development of turnips

Genome-wide gene expression profiles analysis, based on high-throughput RNA sequencing, could facilitate the revelation of quantitative changes in transcript abundance and the identification of candidate genes. We identified a large number of DEGs among different developing stages of tuberous roots of turnips (Fig 1E, S2 Table), implying a high complexity of molecular regulations in tuberous root development.

Tuberous root is a nutrition storage organ. Sucrose and starch, proteins and amino acid, dietary fiber, vitamin, and other relevant molecules are accumulated in the tuberization process [1, 11, 13]. Many metabolic pathways may be actively involved in this process. Based on our genome-wide DEGs analysis, metabolic and biosynthesis of secondary metabolite pathways were the top two pathways with a large number of DEGs significantly enriched, which is consistent with previous studies on the tuberous root of sweet potatoes [16, 21–23], radishes [14], and cassavas [20]. Of these metabolism pathways, starch and sucrose metabolism pathway and phenylpropanoid biosynthesis pathway are the top two evident pathways, which may be active in both tuberous root initiation and the secondary thickening process. In cassavas, glycolysis/gluconeogenesis was identified to be the most evident pathway involved in tuberous root development [20]. In turnips, this pathway was probably not so actively involved, because we did not see a significant enrichment.

Phytohormones play important roles in the regulation of tuberous formation and development [4–7]. Auxin [4–5], CTK [4–5, 7], and JA [7] were reported to be crucial regulators for tuberous initiation, while auxin [4], CTK [6], and ABA [6] probably regulate the secondary thickening growth of tuberous roots. In our study, plant hormone signal transduction pathways were significantly enriched in ES vs. CSS, but not in ES vs. RTS or CSS vs. RTS (Table 2). This suggests that DEGs related to plant hormone signal transduction may play a more important role in tuberous root initiation than in the secondary thickening growth of tuberous roots in turnips. Besides DEGs involved in auxin, CTK and JA signal transduction pathways, DEGs involved in BR, GA, and ethylene signal transduction pathways were also identified in ES vs. CSS (S4 Table), indicating potential roles of these plant hormones in regulating tuberous root initiation in turnips. Furthermore, over half of the DEGs involved in plant hormone signal transduction pathways in ES vs. CSS belong to auxin and CTK pathways, implying the predominant roles of these two phytohormones in regulating tuberous root initiation in turnips.

Several genes related to tuberous development have been identified in previous studies. *MADS* box genes, such as *IbMADS1*, *SRD1*, and *IbAGL17*, were identified to be preferentially expressed in tuberous root of sweet potatoes [4, 8, 48]. In this study, four homologous of *MADS* box genes (*Bra017340*, *Bra004928*, *Bra006051*, and *Bra009913*) were identified to be possibly involved in tuberous root development in turnips. *Bra017340* was more predominantly expressed in CSS libraries than in ES and RTS libraries, indicating a possible positive role of *Bra017340* in tuberous initiation process. *Bra004928* and *Bra006051* were up-regulated in ES vs. RTS, which are likely to be positively involved in the secondary thickness process of tuberous root development in turnips. *Bra009913* were down-regulated in both ES vs. RTS and CSS vs. RTS, implying its negative role in regulating tuberous root development. Expansin (*EXP1*) has been reported to play a negative role in the formation of storage root in sweet potatoes [10]. In our study, the homologous gene *EXP1* (*Bra007864*) and five other Expansin genes (*Bra012218*, *Bra036225*, *Bra028878*, *Bra025372*, and *Bra036499*) were identified to be down-regulated in both ES vs. CSS and ES vs. RTS. Previous studies reported SuSy plays a key role in regulating tuberous root development [12], and homologous of the enzyme are up-regulated in tuberous roots of radishes [14]. In this study, one homologous of *SuSy 3* was up-regulated, and two homologous of *SuSy 6* were down-regulated in ES vs. RTS. No *SuSy* homologous genes were identified differentially expressed in ES vs. CSS. This indicates that SuSy genes probably are involved in tuberous root thickness, but not in tuberous root initiation. New members of *MADS* box genes, expansin genes and *SuSy* were identified for tuberous root development, indicating that high-throughput RNA sequencing is effective in identifying DEGs.

### miRNAs involved in tuberous root development of turnips

miRNAs are important regulators of many important biological processes, such as development, hormone signaling, abiotic stress responses, and pathogen responses [49–51]. They negatively regulate their target gene expression at the post-transcriptional level. Large-scale miRNA expression profiles were analyzed on the modified leafy head development of Chinese cabbage, providing a relatively complete view of the miRNAs involved in the modified leafy head development of *B*. *rapa* [33]. Whether miRNAs are involved in the modified tuberous root development in *B*. *rapa* is still unclear. According to our results, many miRNAs were found differentially expressed in different development stages of tuberous roots in turnips [S6 Table]. In previous studies, it was reported that miR156 and miR172 are involved in tuberous stem development process in potatoes. Overexpression of miR156 reduces the yield of tuberous stem [29], while miR172 overexpression accelerates the tuberization in potatoes [30]. miR156 down-regulates the expression of miR172 by targeting one of the squamosa promoter binding protein-like (SPL) genes, *StSPL9*, a transranscription factor of miR172 [29]. In this study, we found miR156a was down-regulated in the root tuberization process, while miR172a was up-regulated in CSS and RTS compared with ES, which is coincide with that in stem tuberization process in potatoes. miR157 families are predicted to target the *SPL* genes at the same binding sites as miR156 families [52]. In our study, similar to miR156a, the expression of miR157a was down-regulated during the tuberization process. They are likely to play the same roles in regulating tuberous root development in turnips. Furthermore, miR156a, miR157a and miR172a exhibited a relatively high expression level in all samples, and their expression patterns were negatively correlated with all or some of their target DEGs. It indicated that these three miRNAs may play important roles in tuberous root development of turnips. Further studies need to be done to fully understand their functions in regulating tuberous root development.

Gene Ontology analyses of the target genes of the DEMs were similar with those of the DEGs (Fig 2). Although no KEGG pathway was significantly enriched due to the small numbers of the target genes, more genes were clustered in the plant hormone signal transduction pathway in ES vs. CSS and ES vs. RTS, and more were enriched in metabolic pathways in CSS vs. RTS (S9 Table). It supports our hypothesis that plant hormone signal transduction pathway plays more important roles in tuberous root initiation.

Since miRNAs negatively regulate gene expression at the post-transcriptional level, the expression profiles of DEMs are expected to be negatively correlated with the target gene expression profiles. Previous studies have shown that some miRNAs were co-expressed with their target genes [53–55]. We analyzed the expression profiles of the putative target genes of DEMs based on the DEGs sequencing, and found that many miRNA expression profiles were not correlated with their target gene expressions. Similar results were obtained in Chinese cabbage for the modified leafy head development [33]. The results indicate that target gene expression is not regulated by miRNAs alone, but many other factors.

qRT-PCR validation of the six DEMs showed that four DEMs (miR156a, miR157a, miR172a, and novel-mir-208) exhibited the same expression tendency as the original results, while two (miR395b, and novel-mir-108) did not match with those of the original results from the high-throughput sRNA sequencing (S6 Table, Fig 3B). This is probably due to the extremely low expression levels (less than 300 reads were detected in each sample) of the two miRNAs detected in the sRNA sequencing.

## Conclusions

We analyzed both mRNA and miRNA expression profiles of the tuberous root of turnips at different stages based on high-throughput sequencing technology. Our results indicate that a large number of DEGs and DEMs are involved in tuberous root development. According to the DEG analysis, we propose that metabolism is the dominant pathway in both tuberous root initiation and secondary thickening process; the plant hormone signal transduction pathway may play a more important role in regulating tuberous root initiation; and the starch and sucrose metabolism may be more important for the secondary thickening process. Our analyses for DEMs partially supported these hypotheses. Based on the DEM analyses, miR156a, miR157a, and miR172a may play important roles in tuberous root development in turnips. These findings will contribute to our further understanding of the molecular mechanisms of tuberous root development in turnips.

## Supporting Information

S1 Fig Saturation analysis of the six mRNA libraries. A, B, C, D, E, and F were saturation analysis of ES1, ES2, CSS1, CSS2, RTS1, and RTS2 library, respectively.
(TIFF)

S2 Fig Randomness assessments of the six mRNA libraries. A, B, C, D, E, and F were randomness assessments of ES1, ES2, CSS1, CSS2, RTS1, and RTS2 library, respectively.
(TIFF)

S3 Fig Length distribution of sRNAs. (TIFF)

S1 Table qRT-PCR primers for seven DEGs and six DEMs. (XLSX)

S2 Table Genes expressed in each mRNA library. The genes with Log2 ratio were DEGs in the corresponding comparison.
(XLSX)

S3 Table Statistics of differentially expressed genes and miRNAs across all libraries in each replication. (XLSX)

S4 Table Plant hormone signal transduction pathways detected in the study. (XLSX)

S5 Table Distribution of sRNAs among different categories in the six libraries. (XLSX)

S6 Table miRNAs identified in each sRNA libraries. The genes with Log2 ratio were DEGs in the corresponding comparison. Novel miRNAs shown in bold are ones having miRNA* identified in the study.
(XLSX)

S7 Table Bra-miRNAs identified in each sRNA libraries. (XLSX)

S8 Table DEMs and their target gene. Target genes shown in bold red are DEGs with their expression profiles matched up with their corresponding DEMs, while those shown in bold blue are DEGs with their expression profiles not matched up with their corresponding DEMs.
(XLSX)

S9 Table Pathway enrichment analysis of target genes of DEMs detected in both replicates (top three pathways are shown for each comparison). (XLSX)
