# Supplementary figures and images for: Integrative Analysis of mRNA and miRNA Expression Profiles of the Tuberous Root Development at Seedling Stages in Turnips
Source: PLoS One. 2015 Sep 14;10(9):e0137983. doi: 10.1371/journal.pone.0137983 (PMC4569476; doi:10.1371/journal.pone.0137983)

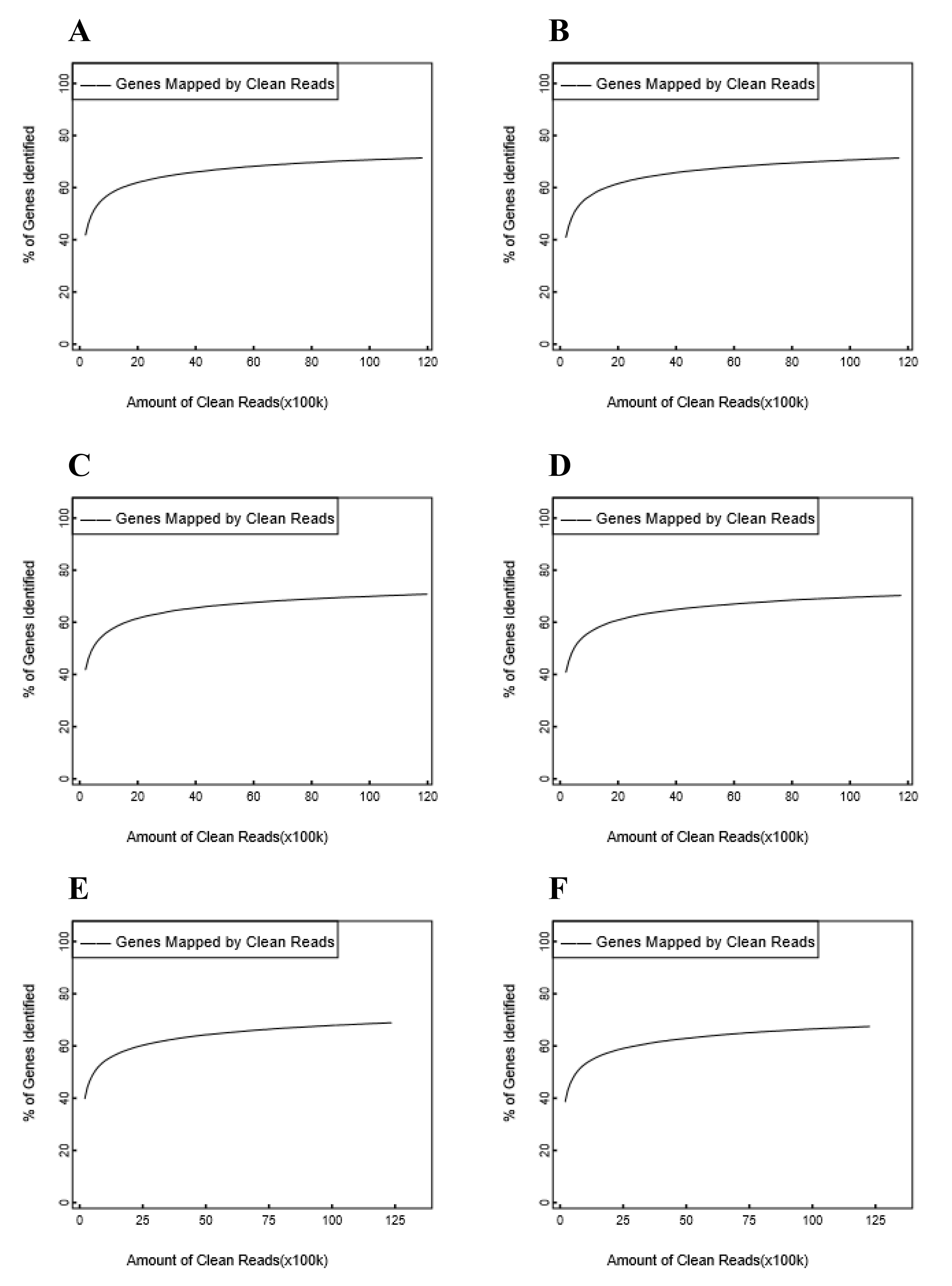

Supplement: S1 Fig — A, B, C, D, E, and F were saturation analysis of ES1, ES2, CSS1, CSS2, RTS1, and RTS2 library, respectively. (TIFF) [file pone.0137983.s001.tiff]

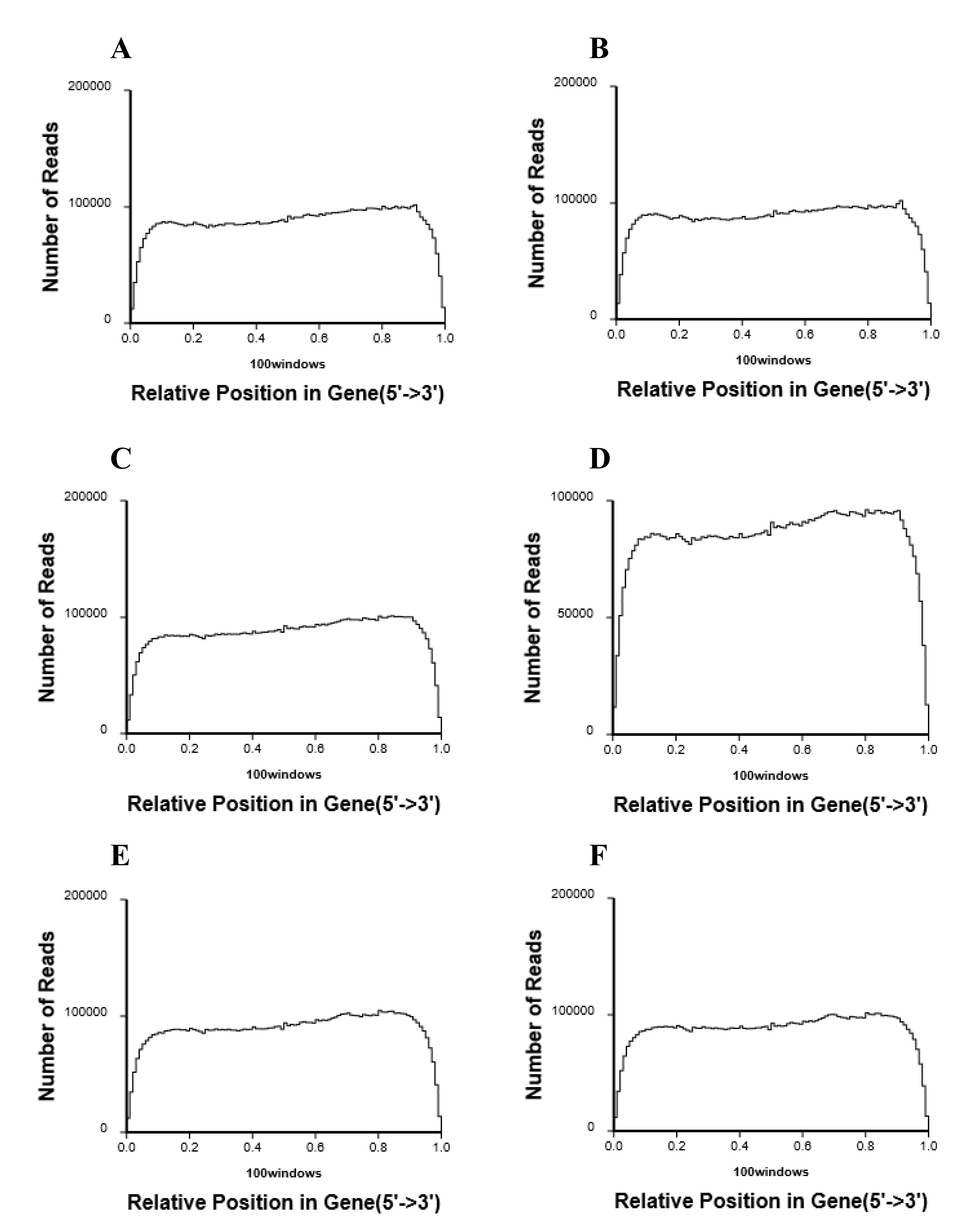

Supplement: S2 Fig — A, B, C, D, E, and F were randomness assessments of ES1, ES2, CSS1, CSS2, RTS1, and RTS2 library, respectively. (TIFF) [file pone.0137983.s002.tiff]

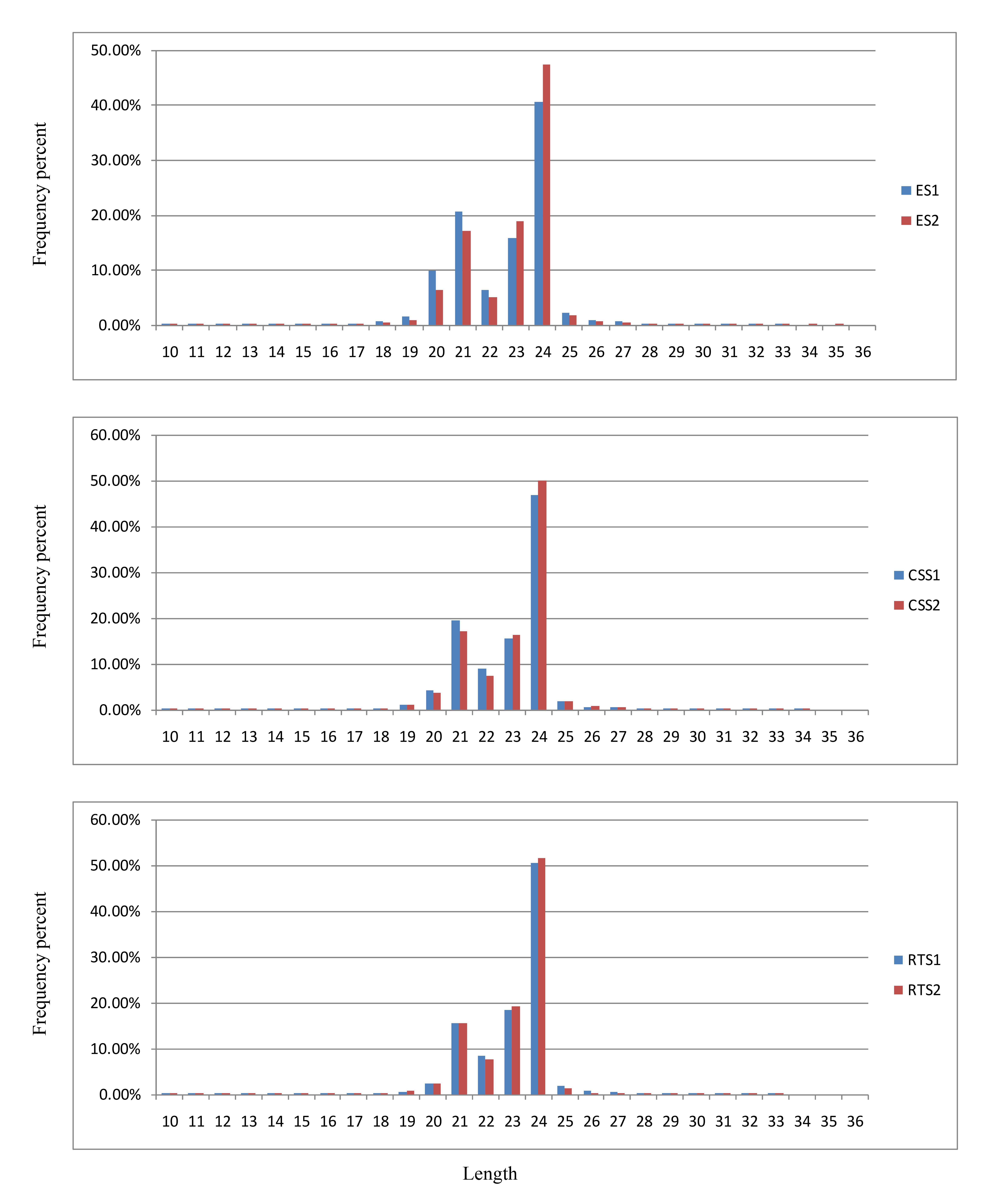

Supplement: S3 Fig — (TIFF) [file pone.0137983.s003.tiff]
